# Modulation of Spinal GABAergic Inhibition and Mechanical Hypersensitivity following Chronic Compression of Dorsal Root Ganglion in the Rat

**DOI:** 10.1155/2015/924728

**Published:** 2015-09-14

**Authors:** Moon Chul Lee, Taick Sang Nam, Se Jung Jung, Young S. Gwak, Joong Woo Leem

**Affiliations:** ^1^Department of Physiology, Yonsei University College of Medicine, 50 Yonsei-ro, Seodaemun-gu, Seoul 120-752, Republic of Korea; ^2^Department of Physiology, Daegu Haany University, 136 Shincheondong-ro, Daegu 706-828, Republic of Korea

## Abstract

Chronic compression of dorsal root ganglion (CCD) results in neuropathic pain. We investigated the role of spinal GABA in CCD-induced pain using rats with unilateral CCD. A stereological analysis revealed that the proportion of GABA-immunoreactive neurons to total neurons at L4/5 laminae I–III on the injured side decreased in the early phase of CCD (post-CCD week 1) and then returned to the sham-control level in the late phase (post-CCD week 18). In the early phase, the rats showed an increase in both mechanical sensitivity of the hind paw and spinal WDR neuronal excitability on the injured side, and such increase was suppressed by spinally applied muscimol (GABA-A agonist, 5 nmol) and baclofen (GABA-B agonist, 25 nmol), indicating the reduced spinal GABAergic inhibition involved. In the late phase, the CCD-induced increase in mechanical sensitivity and neuronal excitability returned to pre-CCD levels, and such recovered responses were enhanced by spinally applied bicuculline (GABA-A antagonist, 15 nmol) and CGP52432 (GABA-B antagonist, 15 nmol), indicating the regained spinal GABAergic inhibition involved. In conclusion, the alteration of spinal GABAergic inhibition following CCD and leading to a gradual reduction over time of CCD-induced mechanical hypersensitivity is most likely due to changes in GABA content in spinal GABA neurons.

## 1. Introduction

Neuropathic pain caused by diseases or injury involving the peripheral or central nervous system has characteristic symptoms of spontaneous burning pain, allodynia (nonpainful becomes painful), and hyperalgesia (painful becomes increasingly painful) [1]. Clinical reports have indicated that more than 60% of neuropathic pain patients were associated with spinal abnormalities including spinal infections and tumors, as well as structural changes of spinal vertebra, such as spinal disc herniation, spinal stenosis, and intervertebral foramen stenosis [2]. Compression of a spinal nerve induced by structural changes of spinal vertebra leads to radicular or low back pain that originates from the lower back and radiates down to the back of the leg and the foot [3]. In addition, about 40% of chronic low back pain patients are shown to have neuropathic pain [4]. Thus, it is possible that peripheral neuropathic pain and radicular pain share a common underlying mechanism.

A rat model of radicular pain that has been developed by chronic compression of the dorsal root ganglion (CCD) demonstrates behavioral signs of neuropathic pain such as mechanical allodynia, hyperalgesia, and enhanced excitability of dorsal root ganglion (DRG) neurons [5–7]. The massive impulses that originate from injured DRG neurons to the spinal cord contribute to altered spinal synaptic plasticity or central sensitization known as the neural mechanisms of hyperalgesia and allodynia. One of the speculated aspects of the underlying mechanisms in spinal central sensitization is the decrease of spinal GABAergic inhibition, or spinal GABAergic disinhibition, caused by the impairment of GABAergic inhibition in the spinal cord. Although this spinal GABAergic disinhibition in neuropathic pain behaviors following peripheral nerve injury has previously been studied, different results are reported. The cell deaths of spinal GABA neurons were observed after injury [8–10]. However, no loss of spinal GABA neurons after injury was detected [11–13]. In addition, an increased GABA level in the spinal dorsal horn was found after injury [14].

The involvement of spinal GABAergic disinhibition in CCD-induced neuropathic pain has not been well studied. The CCD injury model revealed that a gradual recovery of neuropathic pain behavior occurs over time [15]. Thus, one way to verify a spinal GABA involvement in the CCD model is to examine the changes of spinal GABAergic inhibition, which depends on GABA levels and GABA receptor activity, both in the early and later phases following CCD. In the present study, we have investigated first whether the change in the number of GABA neurons as compared with the total neurons in the lumbar spinal dorsal horn does exist following CCD. Second, we have determined the effects of pharmacological activation of spinal GABA receptors on established mechanical hypersensitivity in the early phase, as well as the effects of inhibition of spinal GABA receptors on the reduced hypersensitivity in the later phase.

## 2. Materials and Methods

### 2.1. Animals and Surgery for CCD

A total of 83 male rats (Sprague-Dawley, 180–200 g, Korea) were used in this study and housed at an animal facility with autocontrolled temperature, humidity, and light-dark cycles of 12 hours. All experimental procedures were performed according to the NIH and the Institutional Animal Care and Use Committee of Yonsei University College of Medicine. Chronic compression of the fifth lumbar DRG was performed as previously described [5]. Under deep anesthesia (enflurane, induction 5% and maintenance 2% in mixed oxygen gas), the back skin and muscle were incised to expose the lumbar fifth vertebra and intervertebral foramens. To compress the L5 DRG and nerve root, a sterilized stainless steel rod (0.7 mm diameter and 4 mm length) was inserted in the space of the fifth intervertebral foramen. After injury, the musculature and skin were sutured. A sham operation was performed according to the same procedures; however, the stainless steel rod was not inserted. The postsurgical care was performed with food and water* ad libitum*. Animals were allocated into four groups: those that had received CCD injury (*n* = 26) or sham operation (*n* = 26) 18 weeks earlier and those with CCD one week (*n* = 26) or 8 weeks (*n* = 5) earlier. The sham-operated, 1-week, and 18-week post-CCD groups were subjected to studies of pharmacological responses to GABA-related drugs, in which saline-treated controls were used for stereological cell counting and electrophysiological recordings. The 8-week post-CCD group included animals used for stereological cell counting. All experiments were performed by investigators blinded to animal treatments.

### 2.2. Drugs and Application

The effects of pharmacological activation and inhibition of spinal GABA receptors on mechanical sensitivity and WDR neuronal activity were examined using drugs including GABA-A receptor agonist muscimol (Tocris Cookson, UK) and antagonist (−)-bicuculline methobromide (Tocris Cookson, UK) as well as GABA-B receptor agonist baclofen (Sigma, Saint Louis, MO, USA) and antagonist CGP52432 (Tocris Cookson, UK). The drugs and doses were selected according to previous studies [16–18]. All drugs were dissolved in 0.9% saline and administered intrathecally using a lumbar puncture for studying mechanical sensitivity changes via behavioral assessment; alternatively, drugs were also applied topically on the spinal cord for examining neuronal activity changes via electrophysiological assessment. For intrathecal administration by lumbar puncture, a 26-gauge needle attached to a Hamilton syringe was inserted into the groove between the T13 and L1 vertebrae and carefully advanced into the intervertebral space, with the angle of the needle at about 10 degrees. Proper placement of the needle in the lumber subarachnoid space was confirmed at the time of entry by a sudden loss of resistance and a brief twitch of the muscles of the hip and thigh. Each drug in a volume of 10 *μ*L was administrated slowly over a 30-second period. For topical application, drugs (10 *μ*L) were applied using a Hamilton syringe onto the spinal cord surface near the site where the recording electrode was located. Data were collected at 15 min after drug application, as this time point was empirically determined to be the best for testing during the period of drug efficacy (typically 5–50 min).

### 2.3. GABA Immunohistochemistry

Under deep anesthesia (urethane, 12.5 mg/kg, i.p.), animals were perfused transcardially with heparinized solution-A (50 mM cacodylate acid and 1% sodium metabisulfite), followed by solution-B (100 mM cacodylate acid, 2.5% glutaraldehyde and 1% sodium metabisulfite). After spinal laminectomy, the L4-L5 spinal cord was dissected out, followed by postfixation for 2 h at room temperature. With the L4-L5 spinal cord blocks, transverse sections (40 *μ*m thick) were cut using a Vibratome (VT1000M, Leica, Germany). Two consecutive sections were collected every 15 sections into 24-well plates containing solution-C (50 mM Tris and 1% sodium metabisulfite) for GABA and NeuN immunostaining. In brief, free-floating sections were pretreated in 49% solution-C, 50% methanol, and 1% H_2_O_2_ for 15 min, and then washed in solution-C (3 × 5 min) and blocked in 98% solution-C and 2% normal goat serum for 3 h. Sections were incubated with anti-GABA (1 : 100, AB131, Millipore, Billerica, MA, USA) or anti-NeuN (1 : 400, MAB377, Millipore, Billerica, MA, USA) in a blocking solution for 16 h at 4°C, and then washed in solution-D (50 mM Tris and 145 mM sodium chloride, 3 × 5 min). Sections were then incubated with biotinylated goat anti-mouse IgG (1 : 200) for 1 h. After washing in solution-D (3 × 5 min), sections were incubated with an avidin-biotin peroxidase complex (1 : 200) for 1 h and washed in solution-D (3 × 5 min). For visualization, sections were incubated with DAB substrate kit solution (Invitrogen, Frederick, MD, USA) containing DAB and 0.6% H_2_O_2_ for 5 min, and then followed by dehydration using 80, 90, and 100% ethyl alcohol and xylene. Finally, sections were mounted on slides and fixed with permount mounting media (SP15-100 Toluene Solution UN1294, Fisher Scientific, El paso, Texas, USA) under cover glasses.

### 2.4. Computer Assisted Stereological Analysis

The total number of GABA- and NeuN-immunoreactive (ir) cells was estimated from the immunostained sections (9–12 sections for each cell type) using Computer Assisted Stereological Toolbox (CAST), consisting of an imaging system (Olympus BX-51, Melville, NY, USA) and software (CAST grid version 2.3.1.5, Olympus, Albertslund, Denmark). For counting cells, a section superimposed by the grid moved in stepwise motion to grid intersections along the step length in both *x*-axis and *y*-axis (92 × 92 *μ*m) in laminae I–III so that a particular position of grid intersections was moved to the center of the field of view on a monitor. The section was then viewed under a 100x oil immersion objective on the monitor to set the counting frame, with its size being 20% of the area of the square with a side length of *x*-steps and *y*-steps. The thickness of each section was determined before counting proceeded, and counting was performed with the optical dissector through a 20 *μ*m depth of a counting frame. Among cells that came into focus within the 20 *μ*m height of the optical dissector, only cells inside the counting frame plus those that touched the left and bottom lines of the counting frame were included for cell counts. The estimation of the total number of cells in L4-L5 was performed using this formula: *N* = Σ*Q* × (1/ASF)×(1/SSF)×(1/TSF), where *N* is the estimated total number of cells, Σ*Q* is the number of counted cells, ASF is the area sampling fraction (the area of the counting frame/the area of the sampling grid), SSF is the section sampling fraction (the number of sections sampled for analysis/the number of sections obtained for staining), and TSF is the thickness sampling fraction (the thickness of the counting frame/the thickness of the section) [19].

### 2.5. Behavioral Assessment

Each rat was housed and held 15 min in a transparent acryl box (8 × 8 × 25 cm) on a metal mesh to avoid environmental stress. After accommodation, six applications of the von Frey filament (log unit 3.61–5.46, equivalent to 0.4–26.0 g, North Coast Medical, CA, USA) were applied at the center of the plantar surface of the hind paw; these tests measured paw withdrawal responses through the biting of the filaments, head turning, and changes of body posture. The paw withdrawal thresholds (PWTs) on filament application were determined by the modified up-down testing paradigm [20] using the formula: log⁡⁡  (50% threshold) = *Xf* + *κδ*, where *Xf* is the value of the final von Frey filament (log unit), *κ* represents the correction factors (from a calibration table), and *δ* represents the mean differences of log units between stimuli.

### 2.6. Electrophysiological Assessment

After anesthesia (urethane, 12.5 mg/kg, i.p.), a cannulation was performed for induction and maintenance of skeletal muscular relaxation, with a tracheostomy additionally performed for ventilation. The respiration of each rat was maintained by ventilator (CWE Inc., Ardmore, PA, USA), and CO_2_ levels monitored by CO_2_ analyzer (CWE Inc., Ardmore, PA, USA) were held stable at 3.5%–4.5%. The body temperature of the rat was maintained at about 36-37°C with a thermal blanket. A laminectomy at T13–L2 was performed to expose the lumbar enlargement, and the rat was then fixed on a stereotaxic frame, with the exposed spinal cord covered by mineral oil to prevent dryness and electric insults from the environment. The dura matter, arachnoid membranes, and pia matter were removed. For recoding the neuronal activity, wide dynamic range (WDR) neurons in the L4-L5 spinal dorsal horns were chosen. The reason for this choice is due to the important role that the spinal WDR neurons are known to play in signaling sensory-discriminative components of pain [21, 22]. Furthermore, we have previously observed that there exists a correlation between spinal WDR neuronal responses and nociceptive behavior in a spinal cord injury model [17]. To examine the neuronal excitability, a single carbon filament-filled glass microelectrode (2–4 MΩ) was inserted into the dorsal horn (depths of 100–600 *μ*m below the dorsal surface) using a micropositioner (Narishige, Tokyo, Japan). The characterization of WDR neurons was determined according to response patterns to brushing (using a camel-hair brush), pressing (using a large arterial clip, 100 g force, nonpainful), and pinching (using a small arterial clip, 400 g force, painful) stimuli. The single impulses generated from WDR neurons and evoked for 10 sec with each stimulation were amplified (DAM-80, World Precision Instruments, Sarasota, FL, USA), and these amplified signals were fed to an oscilloscope for monitoring as well as to the data acquisition system (CED 1401 plus, Cambridge Electronic Design, Cambridge, UK) for data analysis. The single impulses that were identical in shape and amplitude were collected using a window discriminator (World Precision Instruments, Sarasota, FL, USA), and the collected signals were analyzed with Spike 2 software (Version 5.0, Cambridge Electronic Design, Cambridge, UK). The total number of impulses evoked by each 10 s stimulus was used for data analysis.

### 2.7. Statistical Analysis

The differences of repeated measurements after a treatment from baseline were analyzed using the Friedman repeated measures ANOVA on ranks followed by Dunnett's test for multiple comparisons. For the comparison of two different groups, the Mann-Whitney rank-sum test for unmatched pairs was used. For the comparison of measurements before and after treatment, the Wilcoxon signed rank test for matched pairs was employed. Differences were considered statistically significant, if *P* < 0.05. Data were expressed as mean ± SE.

## 3. Results

### 3.1. Time Course of Changes in Mechanical Sensitivity of the Hind Paw following CCD

**Figure 1:**
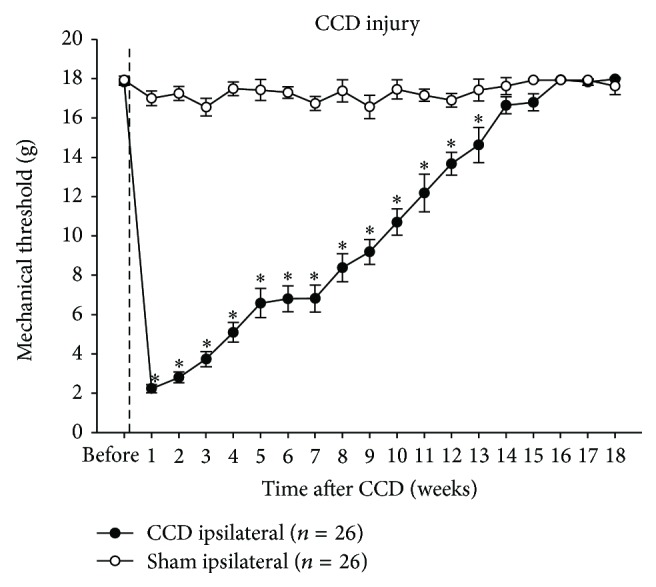
Changes in mechanical sensitivity of hind paws after CCD. A unilateral CCD led to mechanical hypersensitivity of the affected hind paw by showing decreased paw withdrawal thresholds. This hypersensitivity gradually returned to the normal level by post-CCD week 14. ^∗^ *P* < 0.05 when compared with sham-operated rats.

One week after chronic compression of the fifth lumbar DRG (CCD), the paw withdrawal thresholds (PWTs) in the injured side of the hind paw were 2.23 ± 0.21 g and showed a significant decrease when compared with values of pre-CCD (17.83 ± 0.16 g; *P* < 0.05) and sham-operated groups (17 ± 0.37 g; *P* < 0.05). The decreased PWTs gradually recovered to the levels of pre-CCD or sham-operated groups after 14 weeks after the injury (Figure 1, *P* > 0.05). On the contralateral side of the hind paw in CCD groups, no significant changes in PWTs were observed (data not shown).

### 3.2. Changes in GABA-ir Cell Numbers in the Spinal Dorsal Horn following CCD

**Figure 2:**
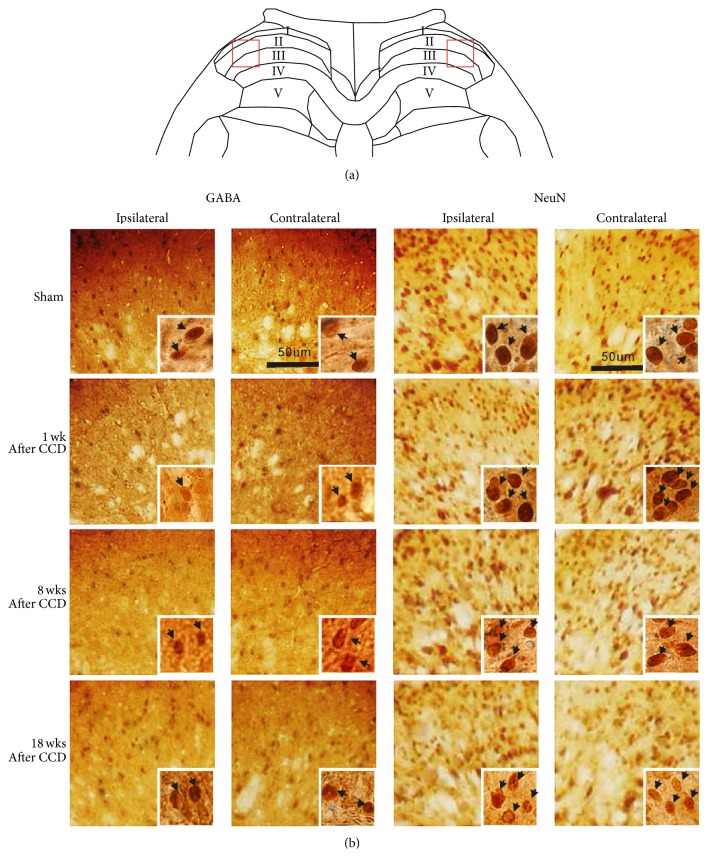
Representative images of GABA-ir and NeuN-ir cells in laminae I–III of the spinal dorsal horn. Photomicrographs of the areas of the squares are presented in (a), which cover the lateral portions of laminae I–III, and are shown in (b). The GABA-ir cells in the ipsilateral laminae I–III appear to be distributed less densely for 1-week and 8-week post-CCD groups than for sham-operated and 18-week post-CCD groups, with no density difference in the contralateral laminae among the four groups. The NeuN-ir cells show no differences in density between both sides of the dorsal horn laminae as well as among all four groups. Typical GABA-ir and NeuN-ir cells are seen in insets at the lower right of each image (indicated by arrows).

Figure 2 presents photographic images for GABA-ir and NeuN-ir immunohistochemistry in the lumbar dorsal horn. The square areas cover the lateral portions of laminae I–III on both sides of the spinal cord, shown in Figure 2(a). For 1-week and 8-week post-CCD groups, GABA-ir cells in ipsilateral laminae I–III appeared less dense than those for the sham-operated and 18-week post-CCD groups; however, no density differences in the contralateral laminae were observed among the four groups (Figure 2(b)). NeuN-ir cells on both sides of laminae showed no density differences among four groups. Typical GABA-ir and NeuN-ir cells are seen in insets at the lower right of each image (indicated by arrows).

**Figure 3:**
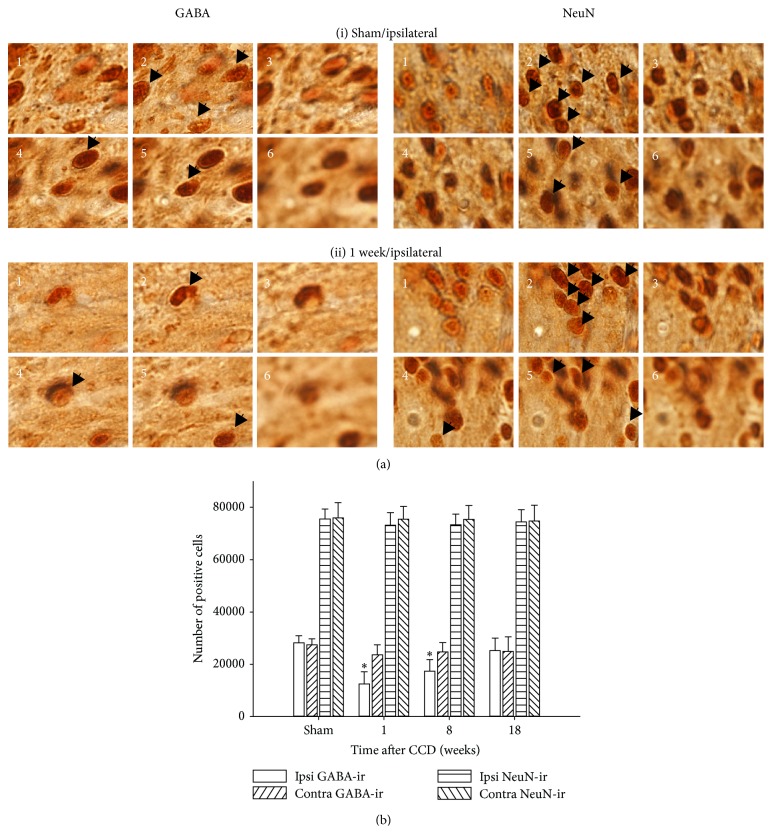
Quantitative analysis of GABA-ir and NeuN-ir cells by stereological cell counting. In (a), images used for stereological cell counts are shown, taken at six consecutive focal planes from the top to the bottom of the 20 *μ*m optical dissector. The dissector contains 5 GABA-ir and 9 NeuN-ir cells for the sham-operated group (i) and 3 GABA-ir and 10 NeuN-ir cells for the 1-week post-CCD group (ii) (indicated by arrows). In (b), the mean numbers of cells estimated by stereological analysis from L4-L5 dorsal horn laminae I–III of individual animals are represented in bar graphs (*n* = 5 rats for each group). The number of GABA-ir cells significantly decreased for both 1-week and 8-week post-CCD groups compared with the sham-operated group, whereas no significant differences in NeuN-ir cell number were seen among all four groups. ^∗^ *P* < 0.05 when compared with sham-treated control.

To illustrate the stereological counting of GABA-ir and NeuN-ir cells, images at six consecutive focal planes from the top to the bottom of the dissector are shown in Figure 3(a). In the examples shown, the dissector for sham-operated group contained 5 GABA-ir and 9 NeuN-ir cells, and the dissector for the 1-week post-CCD group contained 3 GABA-ir and 10 NeuN-ir cells (indicated by arrows). Mean thicknesses of cord sections sampled for stereological counting (10 sections per a rat, five rats per each group) were 26.7, 27.2, 27.6, and 27.3 *μ*m for sham-operated, 1-week, 8-week, and 18-week post-CCD groups, respectively. The total number of cell counts in L4-L5 dorsal horn laminae I–III of each animal was estimated according to the formula described in the Materials and Methods section, and mean values for the four experimental groups are presented in Figure 3(b). The total number of GABA-ir cells significantly decreased (*P* < 0.05) on the ipsilateral side to injury for both 1-week (12,468 ± 4,667) and 8-week (17,357 ± 4,424) post-CCD groups, compared with sham-operated groups (28,159 ± 2,763), with no significant differences on the contralateral side among the four groups. For NeuN-positive cells, however, the total numbers did not show significant differences.

### 3.3. Effects of Activation or Deactivation of Spinal GABA Receptors on Mechanical Sensitivity

**Figure 4:**
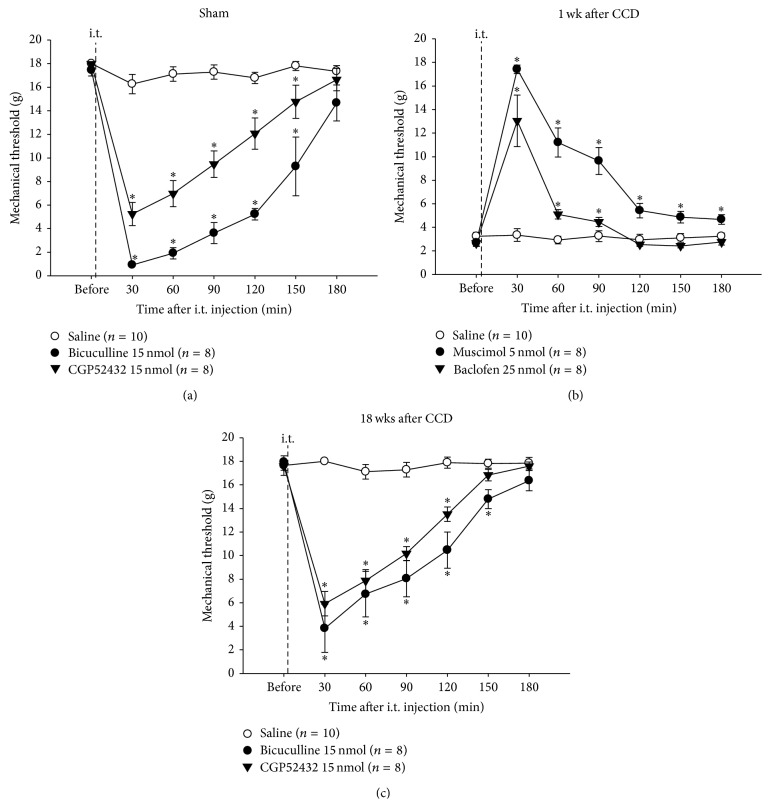
The effects of inhibition or activation of spinal GABA receptors on mechanical sensitivity following CCD. In sham-operated rats (a), an intrathecal (i.t.) administration of bicuculline (15 nmol) or CGP52432 (15 nmol) produced a decrease in the paw withdrawal thresholds (PWTs) for at least 150 min. On 1 week after CCD (b), an i.t. administration of muscimol (5 nmol) or baclofen (25 nmol) reversed the decreased PWTs for longer than 90 min. On 18 weeks after CCD (c), bicuculline (15 nmol) or CGP52432 (15 nmol) resulted in the decrease of PWTs for at least 150 min. ^∗^ *P* < 0.05 when compared with saline-treated control.

In sham-operated animals (Figure 4(a)), an intrathecal (i.t.) administration of bicuculline (15 nmol, *n* = 8) or CGP52432 (15 nmol, *n* = 8) reduced the PWTs maximally at 30 min postdrug (0.92 ± 0.02 g and 5.23 ± 0.98 g, resp.) compared with predrug controls (17.45 ± 0.50 g and 17.98 ± 0.03 g, resp.; *P* < 0.05) as well as with saline-injected controls (17.97 ± 0.02 g; *n* = 10; *P* < 0.05). The reduced PWTs lasted for 150 min. In rats with decreased PWTs on 1 week after CCD (Figure 4(b)), muscimol (i.t., 5 nmol, *n* = 8) or baclofen (i.t., 25 nmol, *n* = 8) reversed the reduced PWTs maximally at 30 min postdrug (17.42 ± 0.37 g and 13.04 ± 2.20 g, resp.) compared with preapplication controls (2.69 ± 0.31 g and 2.66 ± 0.24 g, resp.; *P* < 0.05) as well as with saline-injected controls (3.33 ± 0.54 g; *n* = 10; *P* < 0.05). The reversion lasted for 180 min. In rats with PWTs that had recovered completely to pre-CCD levels on 18 weeks after CCD (Figure 4(c)), bicuculline (i.t., 15 nmol, *n* = 8) or CGP52432 (i.t., 15 nmol, *n* = 8) produced a maximum reduction of PWTs at 30 min postdrug (3.83 ± 2.04 g and 5.91 ± 1.04 g, resp.) compared with predrug controls (17.95 ± 0.23 g and 17.59 ± 0.36 g, resp.; *P* < 0.05) as well as with saline-injected controls (17.64 ± 0.82 g; *n* = 10; *P* < 0.05). The reduced PWTs lasted for 150 min.

### 3.4. Effects of Activation or Deactivation of Spinal GABA Receptors on Neuronal Excitability

**Figure 5:**
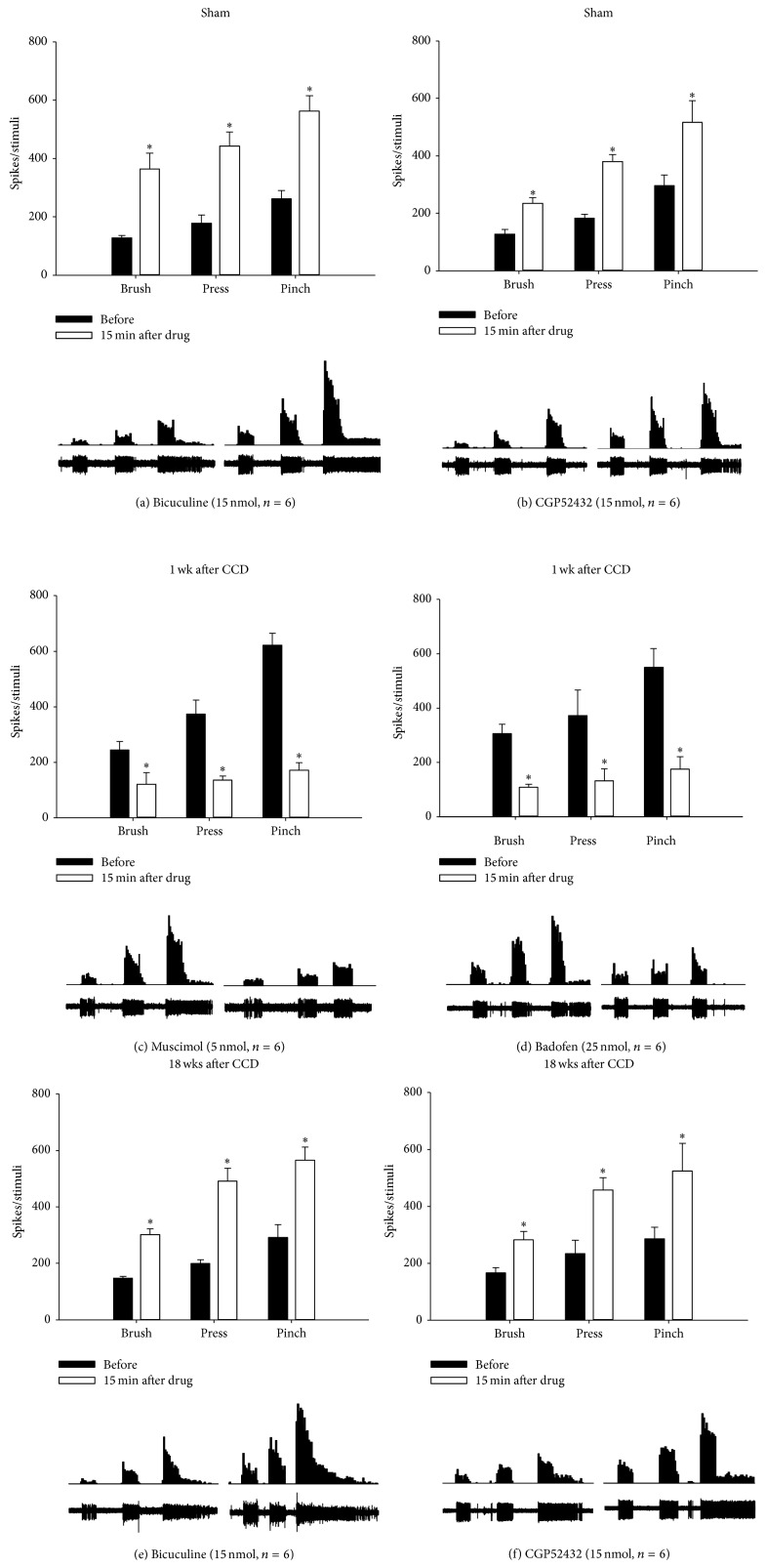
The effects of inhibition or activation of spinal GABA receptors on WDR neuronal activity following CCD. In sham-operated animals, topical application of bicuculline ((a), 15 nmol) or CGP52432 ((b), 15 nmol) enhanced the activity of spinal WDR neurons evoked by brushing, pressing, and pinching stimuli. On 1 week after CCD, topical application of muscimol ((c), 5 nmol) or baclofen ((d), 25 nmol) attenuated the enhanced evoked activity of WDR neurons. On 18 weeks after CCD, bicuculline ((e), 15 nmol) or CGP52432 ((f), 15 nmol) led to an increase in evoked activity of WDR neurons. Data were expressed as the mean number of spike discharges generated from a single WDR neuron evoked by three types of 10 s mechanical stimuli. Below each bar graph, examples of extracellular recordings of stimulus-evoked spike discharges from a single WDR neuron (lower rows) and of peristimulus time histograms for visualizing the rate of spike discharges (upper rows) are shown. ^∗^ *P* < 0.05 when compared with predrug control.

In the sham-operated group (Figures 5(a) and 5(b)), topical application of bicuculline (15 nmol, *n* = 6) significantly increased responses of spinal WDR neurons to brushing, pressing, and pinching stimuli at 15 min postdrug (364 ± 55, 443 ± 48, and 562 ± 53 spikes) compared with predrug controls (128 ± 9, 178 ± 29, and 262 ± 28 spikes; *P* < 0.05). The application of CGP52432 (15 nmol, *n* = 6) also significantly increased such responses (235 ± 20, 380 ± 27, and 517 ± 75 spikes), compared with predrug control (128 ± 16, 183 ± 14, and 297 ± 36 spikes; *P* < 0.05). This antagonism of GABA receptors also enhanced WDR neuronal after discharge activity.

In rats on 1 week after CCD (Figures 5(c) and 5(d)), WDR neuronal responses to the three mechanical stimuli were significantly enhanced under predrug conditions (245 ± 30, 374 ± 50, and 621 ± 44 spikes for premuscimol and 306 ± 35, 373 ± 93, and 550 ± 69 spikes for prebaclofen), compared with those seen in the sham-operated groups (refer to values of predrug controls in Figures 5(a) and 5(b); *P* < 0.05). These enhanced responses were suppressed by topical application of muscimol (121 ± 42, 135 ± 16, and 171 ± 27 spikes; 5 nmol; *n* = 6) and baclofen (109 ± 11, 132 ± 44, and 175 ± 46 spikes; 25 nmol; *n* = 6), respectively. The WDR neuronal after discharge activity seen in rats on 1 week after CCD was also suppressed by this activation of GABA receptors.

In rats on 18 weeks after CCD (Figures 5(e) and 5(f)), WDR neuronal responses to the three mechanical stimuli returned to the sham-control levels (refer to values of predrug controls in Figures 5(a) and 5(b)) as seen under predrug conditions (149 ± 7, 201 ± 14, and 294 ± 45 spikes for prebicuculline and 167 ± 18, 234 ± 47, and 286 ± 41 spikes for pre-CGP52432). These recovered responses were enhanced by topical application of bicuculline (304 ± 21, 494 ± 45, and 567 ± 47 spikes; 15 nmol; *n* = 6) and CGP52432 (283 ± 29, 458 ± 43, and 524 ± 97 spikes; 15 nmol; *n* = 6), respectively. As seen in the sham-control group, this antagonism of GABA receptors resulted in enhancement of WDR neuronal after discharge activity.

## 4. Discussion

Previous reports have suggested that compression of the fifth lumbar DRG caused neuropathic pain in the injured side of the hind paw [3, 23]. In addition, we and others have reported that decreased spinal GABAergic inhibition, or GABAergic disinhibition, contributed to neuropathic pain, following direct damages to the spinal cord and peripheral nerves, respectively [17, 18, 24]. In the present study, we suggest that the decrease of spinal GABAergic inhibition without the decrease of neurons contributes to mechanical hypersensitivity at the hind paw and spinal neuronal hyperexcitability in the early phase following CCD.

Numerous studies have been performed to seek the underlying mechanisms of CCD-induced hyperexcitability in the DRG and spinal nociceptive neurons that lead to neuropathic pain. For instance, ectopic spontaneous and evoked activities of CCD neurons were enhanced by activation of inflammatory mediators via protein kinase A [25, 26], by protease-activated receptor 2 (PAR2) [27, 28], by increased sodium-channel and decreased potassium-channel activity [29], by upregulation of chemokine receptor 2 (CCR2) induced by monocyte chemoattractant protein-1 (MCP-1) [30], and by activation of P2X receptors [31], respectively. However, the implication of spinal GABAergic disinhibition in CCD-induced neuropathic pain has not been well studied.

In previous studies, several possible explanations for spinal GABAergic disinhibition in peripheral nerve injury-induced neuropathic pain have been proposed. The first possibility is a loss of GABA cells after nerve injury. Injury-induced nerve impulses trigger massive releases of glutamate in the spinal dorsal horn, followed by cell excitotoxicity that results in the loss of GABAergic cells, consequently leading to decreased GABAergic inhibitory function [8–10, 32, 33]. However, this hypothesis has been challenged; quantitative analysis has revealed that the number of spinal GABA neurons in rats with peripheral nerve injury is not different from controls [11–13, 34]. In addition, our data showed no changes in the total number of neurons at L4-L5 lumbar dorsal horn laminae I–III following CCD and suggested no decrease of GABA neurons in rats with CCD. The second proposed explanation is the decrease of GABA synthesis. Studies on peripheral nerve injury models have reported that the level of glutamic acid decarboxylase-65 (GAD-65) decreased in the spinal dorsal horn after peripheral nerve injury [8, 10]. Our data, which demonstrate the decreased proportion of GABA neurons to the total NeuN-ir cells at laminae I–III in the early phase after CCD, agree with a proposal of nerve injury-induced decrease of GABA synthesis, as GABA neurons having an undetectable GABA level may be excluded from the cell count. However, it remains for further studies to evaluate whether CCD causes this decrease of GAD activity. The third possibility is the decreased function of GABA receptors via downregulation or decreased binding affinity without any loss of GABA neurons. In support of this theory, previous studies have demonstrated the decrease of GABA-A receptor mRNA levels in primary afferent terminals after spinal nerve ligation injury [35] as well as the decreased binding affinity of GABA-B receptors in the spinal dorsal horn after sciatic nerve transaction injury [36, 37].

However, no loss of spinal GABA-A receptors has been reported in rats with spared nerve injury [34]. Our data indicate that spinal GABA receptor activity remains intact after CCD, since spinal GABA receptor inactivation and activation could influence mechanical sensitivity and neuronal excitability in the early and late phases after CCD. Our data also demonstrate a close correlation between the time-dependent changes of spinal GABA content and mechanical sensitivity following CCD, suggesting an involvement of spinal GABAergic function in CCD-induced neuropathic pain. In the early phase after CCD, spinally applied GABA receptor agonists decreased CCD-induced mechanical hypersensitivity, suggesting a reduction of spinal GABAergic inhibition. After the return of mechanical sensitivity to normal levels in the late phase, spinally applied GABA antagonists regenerated mechanical hypersensitivity, suggesting a regain of spinal GABAergic inhibition. We postulate the feasible mechanisms of time-dependent changes in spinal GABAergic inhibition after CCD as follows. The DRG compression induced by a stainless steel rod insertion into the intervertebral foramen is the primary cause for developing mechanical hypersensitivity of the hind paw. In the early phase after CCD, DRG compression-induced enhancement of sensory input triggers an increase of GABA release in the spinal cord, where the balance between excitation and inhibition is usually maintained. The increased spinal GABA release may lead to the decreased GABA synthesis in spinal GABA neurons, which results in decreased spinal GABAergic inhibition, thus producing mechanical hypersensitivity. In the later phase, however, the increased spinal GABA synthesis may occur so that spinal GABAergic inhibition is regained to reduce the CCD-induced mechanical hypersensitivity.

Since our knowledge of spinal synaptic circuits that include inhibitory (mostly GABAergic/glycinergic) and excitatory (mostly glutamatergic) interneurons is limited, it is difficult to explain why spinal GABAergic neurons are vulnerable to CCD. As proposed in the gate control theory of pain [38], inputs from nociceptive and low-threshold (LT) primary afferents converge onto a dorsal horn projection neuron for pain signal transmission to the brain. In this proposal, the LT input also activates an inhibitory interneuron that produces postsynaptic inhibition of the projection neuron and the presynaptic inhibition of LT input. In addition, the nociceptive input to the projection neuron is disynaptic through an excitatory interneuron. Although the projection neuron responds to both nociceptive and LT inputs, the effectiveness of the LT input is normally reduced by the inhibitory interneuron activity. If this inhibitory action is disrupted, the LT input leads to stronger excitation of the projection neuron, which has been shown in this study. A previous study has revealed a high incidence of spontaneous ectopic discharge that is generated after CCD from large-sized DRG neurons of the injured ganglion [39]. This observation suggests that inhibitory transmission is more affected than excitatory transmission in spinal synaptic circuits following CCD, because of excessive ectopic discharge input from the injured LT afferents. Moreover, GABAergic interneurons outnumber glycinergic interneurons, 35% versus 18% of the total neuronal population, in laminae I–III of the dorsal horn [40]. Thus, CCD-induced ectopic discharge of LT afferents would lead to a loss for more GABAergic inhibition than glycinergic inhibition.

It has been reported that the restoration of lumbar intervertebral foramen space improves mechanical hypersensitivity and that decompression of the DRG reduces compression-induced decreases of DRG neurons [41]. Thus, it is possible to expect that gradual attenuation of CCD-induced mechanical hypersensitivity is due to the recovery of damaged CCD neurons by the gradual widening of the intervertebral foramen over a period of time after injury. In clinical data, 60% of neuropathic pain patients who had undergone surgery to enlarge narrowed foramens show attenuation of neuropathic pain within a year [42]. Taken together, the recovery of damaged peripheral nerves and subsequent restoration of spinal GABAergic inhibition are critical for the improvement of chronic neuropathic pain following CCD.

## 5. Conclusions

CCD modulates spinal GABAergic inhibitory function. In the early phase after CCD, spinal GABAergic inhibition is reduced via the decrease of GABA content in GABA neurons, resulting in mechanical hypersensitivity in the hind paw and spinal neuronal hyperexcitability. In the later phase, GABAergic inhibition level is restored to normal via recovery of GABA content, leading to alleviation of such mechanical hypersensitivity and neuronal hyperexcitability. Therefore, the control of spinal GABA levels could be a useful tool for the treatment of neuropathic pain following CCD.

## Highlights

- GABA-ir neurons were decreased without changes in total neuron numbers on the CCD injury side in the early phase, whereas there were no changes in GABA-ir neurons in the late phase.
- Mechanical hypersensitivity and spinal WDR neuronal hyperexcitability developed on the CCD injured side in the early phase, which returned to normal in the late phase.
- GABA receptor agonists reduced CCD-induced mechanical hypersensitivity and neuronal hyperexcitability in the early phase.
- GABA receptor antagonists reinduced mechanical hypersensitivity and neuronal hyperexcitability in the late phase.
